# County-level spatial heterogeneity and environmental correlates of human echinococcosis in Xizang, China

**DOI:** 10.1371/journal.pone.0362680

**Published:** 2026-11-10

**Authors:** Jiajia Ai, Lamu Ciren, Quzhen Gongsang, Lamu Suolang, Xiaoyao Ji, Jiakun Wang, Yangjin Baima, Zhaohui Luo, Zhuoma Bianba

**Affiliations:** 1 Xizang Center for Disease Control and Prevention, Lhasa, China; 2 NHC Key Laboratory of Echinococcosis Prevention and Control, Lhasa, China; Xinjiang Medical University Affiliated First Hospital, CHINA

## Abstract

**Background:**

Human echinococcosis remains a major zoonosis in Xizang, China, where marked climatic, topographic and landscape gradients may be associated with spatial variation in disease prevalence. We investigated county-level spatial patterns of overall human echinococcosis, cystic echinococcosis (CE) and alveolar echinococcosis (AE), and their environmental associations.

**Methods:**

Surveillance data from 74 counties during 2019–2023 were analysed. Because usable surveillance data were unavailable for 32 counties in 2022, that year was excluded from the spatial and generalized additive mixed model (GAMM) analyses. Global and local Moran’s I assessed spatial autocorrelation and clustering. Tweedie GAMMs evaluated nonlinear environmental associations and pairwise interactions, incorporating a county-level random intercept, year as a categorical fixed effect, and a two-dimensional spatial smooth.

**Results:**

Among 510,063 individuals screened, 2,449 cases were detected, including 2,214 CE and 71 AE cases. Mean prevalence was 0.48% overall and 0.43% for CE; AE prevalence was 13.92 per 100,000. Overall human echinococcosis and CE were concentrated in western and northern Xizang, with high–high clusters mainly in the west, whereas AE clustered focally in the northeast. Overall prevalence showed an inverse association with mean annual temperature and a significant interaction between mean annual relative humidity and lowest elevation. CE prevalence showed a nonlinear association with mean annual relative humidity and a significant interaction between mean annual temperature and surface water coverage percentage. In the simplified AE model, lowest elevation showed an approximately linear positive association with prevalence; the AE-specific findings were considered exploratory.

**Conclusions:**

Echinococcosis in Xizang shows pronounced subtype-specific spatial heterogeneity and distinct county-level environmental correlates. These ecological associations should not be interpreted as direct causal effects. Environmental information may complement host, population, behavioral, surveillance, and intervention-related data when identifying areas that warrant enhanced surveillance and further investigation.

## Introduction

Echinococcosis is a globally distributed zoonotic parasitic disease caused by the larval stages of Echinococcus tapeworms. Two species of major medical and public health importance are *Echinococcus granulosus* and *Echinococcus multilocularis*, which lead to cystic echinococcosis (CE) and alveolar echinococcosis (AE), respectively. Both diseases pose significant threats to human health [1]. Their transmission depends on specific ecological cycles involving definitive and intermediate hosts: CE is typically associated with a dog–ungulate host system, while AE involves a sylvatic cycle of wild canids and small mammals [2]. Humans become infected through accidental exposure to environments contaminated with parasite eggs [3,4]. Echinococcosis poses a serious global public health challenge. The global burden of CE is estimated at 1,009,662 disability-adjusted life years (DALYs) annually [5], while AE accounts for an estimated 666,433 DALYs each year [6].

In China, the burden of echinococcosis has shown an upward trend in recent years, with an almost 5.6-fold increase from 2004 to 2020 (from 180 to 1,000 person-years) [7]. Approximately 98.12% of CE cases in China are concentrated in seven provincial-level regions: Xizang, Qinghai, Xinjiang, Ningxia, Gansu, Sichuan, and Inner Mongolia. AE cases are even more geographically concentrated, with 99.07% occurring in just five regions: Qinghai, Xizang, Sichuan, Ningxia, and Xinjiang [8].

Xizang, located in southwestern China, occupies a substantial part of the Qinghai–Tibet Plateau, the world’s highest and largest plateau, with an average elevation exceeding 4,000 meters. Xizang also has the largest surface water area among all administrative regions in China. Its vegetation exhibits a distinct southeast-to-northwest geographical gradient, transitioning from forests to grasslands and eventually to deserts. Xizang contains the largest expanse of grassland in China and is one of the country’s key pastoral regions. The climate of Xizang is unique and highly diverse due to the combined influences of topography, geomorphology, and atmospheric circulation. Overall, the region is characterized by a cold and arid climate in the northwest and a warm and humid climate in the southeast. This distinctive natural environment may be associated with the high prevalence and spatial heterogeneity of echinococcosis observed in the region. According to a national survey, Xizang had the most severe endemicity of echinococcosis in China, with a total prevalence of 1.66% reported in 2016 (1.45% CE and 0.20% AE), ranking highest nationwide [9]. Therefore, Xizang constitutes a key target region for echinococcosis control efforts in China.

To characterize the environmental correlates of this spatial heterogeneity, we conducted a systematic county-level analysis of associations between natural environmental characteristics and human echinococcosis prevalence. A growing body of ecological research has reported associations between natural environmental characteristics and the prevalence or spatial distribution of echinococcosis [10,11]. For example, a study conducted in Ningxia, western China, reported that the human seroprevalence of CE was significantly associated with summer and winter precipitation, landscape fragmentation indices, and the areas covered by forest, shrubland, water bodies, and bare/artificial surfaces. Meanwhile, the seroprevalence of AE showed significant correlations with summer and winter precipitation, landscape fragmentation, and the extent of shrubland and water bodies [12]. Similarly, another study in western China found a positive correlation between human CE prevalence and the proportion of grassland area [13]. Subsequent investigations have reported associations of higher elevation and extensive grassland coverage with higher AE prevalence [14], while CE prevalence exhibits nonlinear associations with summer NDVI [15]. Consistent with these findings, a study conducted in Germany also reported that AE prevalence tends to increase with elevation [16]. Further research has identified a range of factors positively associated with CE prevalence, including elevation, pasture area, whereas forest area appears to have a negative association [17]. Moreover, variables such as distance to water bodies and elevation have shown complex nonlinear associations with CE prevalence [18].

Although surveillance data from 2019 to 2023 are now available, no study has yet examined the spatial heterogeneity of echinococcosis in Xizang during this period, nor have the natural environmental correlates of such heterogeneity been systematically characterized. To fill these critical gaps, we employed an integrated approach combining spatial epidemiology with nonlinear modeling to investigate the spatial patterns of human echinococcosis in Xizang and their associations with natural environmental characteristics. Using high-resolution, county-level datasets that captured fine-scale landscape and climatic features, we combined the spatial gradient modeling strengths of Geographic Information Systems (GIS) with the nonlinear response analysis capability of generalized additive mixed models (GAMMs). This approach enabled us to characterize potentially nonlinear associations between natural environmental characteristics and disease prevalence while accounting for spatial and repeated county-level structure. First, GIS was used to construct gradient maps of prevalence across counties, revealing spatial distribution patterns and hotspot areas of echinococcosis. Second, a series of natural environmental variables, including geographical and meteorological indicators were selected as candidate environmental predictors. Finally, three separate GAMMs were developed to model the prevalence of overall echinococcosis, CE, and AE, respectively. For overall echinococcosis and CE, predefined candidate models were compared using the Akaike Information Criterion (AIC) to assess their relative support, whereas the AE model was specified parsimoniously on the basis of epidemiological and ecological evidence because of the sparse AE data. This study provides county-level ecological evidence that may inform surveillance prioritization and future integrated investigations incorporating environmental, host, and anthropogenic factors in Xizang.

## Materials and methods

### Data collection

#### Prevalence of echinococcosis.

County-level surveillance data on human echinococcosis from 2019 to 2023 were obtained from the Echinococcosis Surveillance System of Xizang. Surveillance was conducted using a standardized active population-screening protocol. In each county (district), one administrative/natural village or several adjacent villages were randomly selected as surveillance site(s) using a cluster-sampling approach. Within each selected as surveillance sites through cluster sampling., residents aged ≥2 years were randomly sampled for echinococcosis screening, with at least 1,000 individuals screened per site. Villages selected as surveillance sites were not eligible for reselection within the subsequent five years. Case diagnosis was based on the Diagnostic Criteria for Echinococcosis (WS 257–2006), which served as the uniform diagnostic standard across all surveillance sites. For each county-year observation, surveillance prevalence was calculated as the number of detected cases divided by the number of individuals screened. Overall echinococcosis, CE, and AE prevalence were calculated separately according to case classification. Overall echinococcosis and CE prevalence were expressed as percentages, whereas AE prevalence was expressed per 100,000 individuals screened because of the relatively small number of AE cases. Because usable county-level surveillance data were unavailable for 32 of the 74 counties (43.2%) in 2022, these counties are shown as “Not known” in the descriptive maps, data from that year were excluded from the spatial autocorrelation and GAMM analyses. These analyses therefore included data from 2019, 2020, 2021, and 2023.

### Geographical landscape factors

Data on forest coverage percentage (FCP), grassland coverage percentage (GCP), and surface water coverage percentage (SWP) were derived from 30-meter resolution land cover data provided by the National Earth System Science Data Center (https://www.geodata.cn). The proportions of each land cover type within the total area of each county were calculated accordingly.

Mean elevation (ME), lowest elevation (LE), and highest elevation (HE) data were obtained from the National Geomatics Center of China (https://www.ngcc.cn), with a spatial resolution of approximately 30 m.

Normalized difference vegetation index (NDVI) values were extracted from MODIS MOD13A3 data, downloaded via the NASA earthdata platform (https://search.earthdata.nasa.gov).

### Meteorological factors

Data on mean annual temperature (T), mean annual wind speed (WS), mean annual daily precipitation (AP), and mean annual relative humidity (RH) were obtained from the ERA5 reanalysis dataset provided by the European Centre for Medium-Range Weather Forecasts (ECMWF) under the Copernicus Climate Change Service (C3S) Climate Data Store (CDS) (https://cds.climate.copernicus.eu/datasets/reanalysis-era5-land). ERA5-Land has a native spatial resolution of approximately 9 km and is distributed through the Climate Data Store on a regular 0.1° × 0.1° latitude–longitude grid, with an hourly temporal resolution.

### Analysis methods

#### Spatial autocorrelation analysis.

Global spatial autocorrelation evaluates whether echinococcosis prevalence exhibits an overall clustered or dispersed pattern across the study region [19]. We first applied Global Moran’s I to test the presence of overall spatial clustering.

Local spatial autocorrelation is then used to identify where clusters occur. Local Moran’s I quantifies the association between each observation and its neighbors and classifies locations into four types: high–high (hot spots), low–low (cold spots), high–low, and low–high (spatial outliers) [20,21]. Local indicators of spatial association (LISA) cluster maps were produced to visualize these local patterns.

The formulas for the global and local Moran’s I statistics are as follows [22,23]:


$$\begin{array}{c} I=\frac{n\sum_{i=\text{1}}^{n}\sum_{j=\text{1}}^{n}w_{ij}\left(x_{i}-\overset{―}{x}\right)\left(x_{j}-\overset{―}{x}\right)}{\sum_{i=\text{1}}^{n}\sum_{j=\text{1}}^{n}w_{ij}{\left(x_{i}-\overset{―}{x}\right)}^{\text{2}}} \end{array}$$ (1)

$$\begin{array}{c} I_{i}=\frac{\left(x_{i}-\overset{―}{x}\right)}{S^{\text{2}}}\sum_{j=\text{1}}^{n}w_{ij}\left(x_{j}-\overset{―}{x}\right),\text{\textasciitilde{}}S^{\text{2}}=\frac{\sum_{j=\text{1},j\neq i}^{n}w_{ij}{\left(x_{j}-\overset{―}{x}\right)}^{\text{2}}}{n-\text{1}} \end{array}$$ (2)

In equations (1) and (2), n is the number of counties, *x*_*i*_ and *x*_*j*_ are the autocorrelation indices from units with indices *i* and *j*, respectively, $\overset{―}{x}\;$is the mean value, and *w*_*ij*_ is the spatial weight matrix. If unit *i* is adjacent to unit *j*, then *w*_*ij*_ = 1; if they are not adjacent, then *w*_*ij*_ = 0. In this study, all spatial autocorrelation analyses were conducted using the Spatial Autocorrelation tools in ArcGIS 10.8 (ESRI, Redlands, USA).

### Statistical modeling: GAMM with Tweedie family for zero-inflated prevalence

To explore the nonlinear relationships between echinococcosis prevalence and multiple natural environmental factors, this study employed GAMM. A GAMM is an extension of the generalized additive model that incorporates random effects, allowing for simultaneous modeling of nonlinear relationships and hierarchical or correlated data structures [24]. The model uses smooth functions, such as splines, to flexibly characterize potentially nonlinear associations between predictors and the response variable. A county-level random intercept was included to account for repeated observations within counties [25,26], while year was modeled as a categorical fixed effect to account for common temporal differences among the analyzed years. County-level echinococcosis prevalence was treated as a semi-continuous response with exact zeros and a right-skewed continuous distribution among positive values. Accordingly, GAMMs with a Tweedie response distribution (compound Poisson–Gamma) and a log link function was fitted. The power index was restricted to 1 < p < 2, allowing the distribution to accommodate both zero and positive continuous values. For each outcome, p was selected by grid search at increments of 0.05, with the value yielding the highest model log-likelihood retained for subsequent analyses. The power index p was estimated by maximizing the log-likelihood function within this range [24,27]. This specification allows nonlinear environmental associations to be modeled while accounting for repeated county-level observations, common differences among study years, and broad-scale residual spatial variation.

The model can be expressed as:


$$\begin{array}{c} Y_{i}\sim Tweedie\left(\mu_{i},\phi ,p\right) \end{array}$$

$$\begin{array}{c} og\mu_{i}=\beta_{\text{0}}+\sum_{j=\text{1}}^{q}s\left(X_{ij},k\right)+s\left({lon}_{i},{lat}_{i},bs{=}^{\prime }tp^{\prime }\right)+\;s\left({County}_{i},bs{=}^{\prime }re^{\prime }\right)+\gamma_{{Year}_{i}} \end{array}$$ (3)

Where $Y_{i}$denotes the response variable, representing the echinococcosis prevalence for the *i-th* county–year observation. $Y_{i}\;$is a non-negative semi-continuous outcome characterized by a discrete probability mass at zero and a continuous positive density for non-zero observations. $\mu_{i}$represents the conditional mean of $Y_{i}$; ϕ and p are the dispersion and power parameters of the Tweedie family, respectively, governing the mean–variance relationship $\text{Var}(Y_{i})=\phi \;\mu_{i}^{p}$. The logarithmic link function ensures that the predicted mean $\mu_{i}\;$remains strictly positive and linearizes the relationship between predictors and the expected value. $\beta_{\text{0}}\;$is the intercept term, and $s(X_{ij},k)\;$denotes a smooth function of the j-th predictor $X_{ij}$. A basis dimension of k = 6 was specified for the smooth environmental terms in the overall echinococcosis and CE models. Given the limited number of AE cases, a more restrictive basis dimension of k = 4 was used for the AE model to limit smooth complexity. The adequacy of the specified basis dimensions was assessed using the basis-dimension diagnostics implemented in gam.check() in the mgcv package. q indexes the q natural environmental covariates included in the model. $s\left(lon_{i},lat_{i},bs{=}^{\prime }tp^{\prime }\right)\;$specifies a two-dimensional thin-plate spline based on county centroid longitude and latitude was included to account for broad-scale residual spatial variation among counties, while $s(County_{i},bs{=}^{\prime }re^{\prime })$ represents a county-level random-effect smoother accounting for unobserved heterogeneity and repeated observations across counties. $\gamma_{{\text{Year}}_{i}}$ represents the categorical fixed effect of year, accounting for common temporal differences among the analyzed years. Thin-plate spline smoothers were applied for all smooth terms, with the degree of smoothness determined by the estimated degrees of freedom (edf), where higher edf values correspond to more flexible and less penalized functions [28].

In this study, county-level human echinococcosis prevalence was defined as the response variable, while natural environmental variables served as candidate environmental predictors. Prior to model construction, multicollinearity diagnostics were conducted among candidate environmental predictors. The Spearman’s rank correlation coefficient (*|r*_s_*|*) and variance inflation factor (VIF) were used to detect potential multicollinearity. Variables with *|r_s_|* ≥ 0.70 or VIF > 10 were excluded to ensure model stability and interpretability [29,30]. When a predictor was excluded, the correlation structure and VIFs were reassessed among the remaining predictors to confirm that the retained variable set did not exceed the prespecified thresholds. The remaining variables were incorporated into a GAMM framework to explore the nonlinear relationships between natural environmental factors and echinococcosis prevalence. A main-effects GAMM including all retained environmental predictors as smooth terms was first fitted. Pairwise nonlinear interactions were then evaluated one at a time by adding each bivariate interaction to the main-effects model while retaining the corresponding main effects. The main-effects model and single-interaction candidate models were compared using AIC, and the model with the lowest AIC within this predefined candidate set was retained for further evaluation. Given the limited number of AE cases, the AE-specific analysis used a more parsimonious model restricted to a prespecified set of environmental predictors selected on the basis of prior epidemiological and ecological evidence, without interaction terms. In-sample model fit was assessed using adjusted R² and deviance explained. The adequacy of the final GAMM was further examined through residual diagnostics, including Q–Q plots, histograms of deviance residuals, and scatter plots of residuals versus fitted values. These diagnostics were used to assess residual distribution, normality, and heteroscedasticity, thereby providing a comprehensive evaluation of model suitability and robustness [31]. To quantitatively assess remaining spatial dependence after model fitting, Moran’s I was additionally calculated for the deviance residuals separately for each study year using the same row-standardized Queen-contiguity spatial weights as in the primary spatial autocorrelation analysis. To assess the out-of-sample performance of the final GAMMs, five-fold grouped cross-validation was performed at the county level. All repeated observations from the same county were assigned to the same fold to avoid information leakage between the training and validation sets. In each iteration, the model was fitted using four folds and evaluated on the remaining fold. Predictive performance was summarized using root mean squared error (RMSE) and mean absolute error (MAE). All analyses were performed in R software (version 4.2.1).

### Sensitivity analyses

Several sensitivity analyses were performed to assess the dependence of the main findings on key analytical specifications. First, Global and Local Moran’s I analyses were repeated using an alternative k-nearest-neighbor (kNN) spatial weights matrix. The resulting global spatial patterns were compared with those from the primary analysis in terms of the direction and statistical significance of spatial autocorrelation, whereas local results were compared according to the number and classification of significant county-level spatial patterns. Second, after selecting the Tweedie power parameter within 1 < p < 2, the final GAMMs were refitted using two alternative power values while keeping the model structure unchanged; the direction and shape of the principal smooth associations and interaction surfaces were then compared across specifications. Third, because AE cases were sparse and unevenly distributed across years, temporal sensitivity was assessed using leave-one-year-out analyses. The final AE model was refitted after excluding each study year in turn, and the resulting smooth associations were compared with the primary model using edf, *F* statistics, *P* values, Pearson correlations, and shape RMSE.

## Results

### Prevalence of echinococcosis

Between 2019 and 2023, a total of 510,063 individuals were screened for echinococcosis across 74 counties in Xizang. A total of 2,449 cases were detected, including 2,214 cases of CE, 71 cases of AE, and 164cases of mixed or unclassified types. The average annual prevalence rates for overall echinococcosis, CE, and AE were 0.48%, 0.43%, and 13.92 per 100,000 population, respectively (Table 1).

**Table 1:** Cases and prevalence of echinococcosis in Xizang Autonomous Region, 2019-2023.

Year	Overall		CE		AE	
	Cases	Prevalence/%	Cases	Prevalence/%	Cases	Prevalence/100,000
**2019**	536	0.32	483	0.29	7	4.23
**2020**	653	0.6	613	0.57	7	6.47
**2021**	533	0.55	434	0.45	53	54.8
**2022**	254	0.49	236	0.45	0	0
**2023**	473	0.54	448	0.51	4	4.57
**Total**	2449	0.48	2214	0.43	71	13.92

The 2022 surveillance data were incomplete because usable county-level records were unavailable for 32 of 74 counties. The values shown for 2022 therefore represent available surveillance records only and were not included in the spatial autocorrelation or GAMM analyses. Overall echinococcosis and CE prevalence are expressed as percentages (%), whereas AE prevalence is expressed per 100,000 individuals screened because of the small number of AE cases. All prevalence measures were calculated using the number of individuals actually screened in the corresponding year as the denominator.

### Spatial distribution of echinococcosis

The county-level prevalence of overall human echinococcosis in Xizang (2019–2023) exhibited a distinct spatial distribution pattern, characterized by pronounced geographic disparities and marked temporal fluctuations. Spatially, counties with higher observed prevalence were predominantly located in western and northern Xizang. Conversely, counties in the south-central part of the region consistently exhibited low prevalence rates (Fig 1).

**Fig 1:**
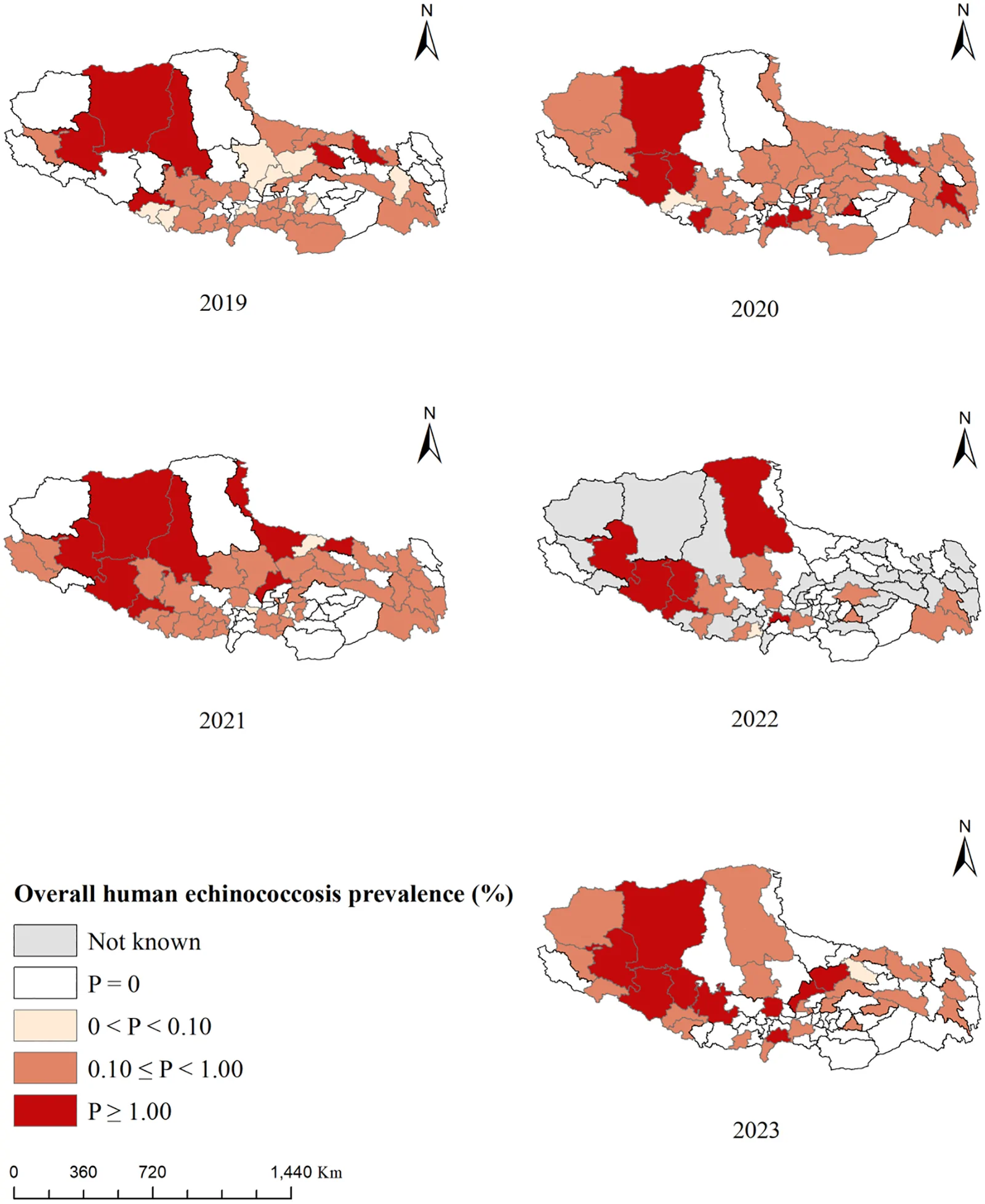
Spatial distribution of overall human echinococcosis prevalence at the county level in Xizang Autonomous Region. The map is based on the standard map with review number GS (2023) 2767 downloaded from the Standard Map Service website of the Ministry of Natural Resources (http://bzdt.ch.mnr.gov.cn/), with no modifications to the base map. Areas shown in grey and labeled “Not known” indicate counties without usable surveillance data. In 2022, this applied to 32 of 74 counties (43.2%).

Temporally, prevalence peaked in 2020, during which Zhongba County recorded the highest rate at 13.60%, and nine counties reported prevalence rates ≥ 1.00%. Although the epidemic subsided after 2021, a slight rise was observed in 2023.

Subtype-specific analysis indicated that the spatial distribution of CE closely resembled the overall prevalence pattern (S1 Fig in S1 File). In contrast, AE exhibited a more localized distribution, with cases concentrated in northeastern Xizang, encompassing a total of 13 counties. AE prevalence peaked in 2021, demonstrating a markedly different temporal trend compared to overall prevalence. (S2 Fig in S1 File).

### Cluster analysis of echinococcosis

Global spatial autocorrelation analysis indicated that overall human echinococcosis prevalence in Xizang exhibited significant positive global spatial autocorrelation in 2023 (Moran’s I = 0.061, *P* = 0.048; Table 2). Similarly, CE prevalence showed positive global spatial autocorrelation in 2019 and 2021 (Moran’s I = 0.082, *P* = 0.042; Moran’s I = 0.087, *P* = 0.032, respectively; S1 Table in S1 File). AE prevalence also demonstrated significant global spatial autocorrelation in 2019 and 2021 (Moran’s I = 0.068, *P* = 0.045; Moran’s I = 0.032, *P* = 0.038, respectively; S2 Table in S1 File). These results indicate that significant global spatial autocorrelation was detected only in selected years and was not consistently present throughout the study period.

**Table 2:** Results of global spatial autocorrelation for overall human echinococcosis prevalence in Xizang Autonomous Region, 2019–2023.

Year	Moran’s I	Z-Score	*P*	Pattern
**2019**	0.049	1.303	0.193	Not clustered
**2020**	0.009	0.582	0.561	Not clustered
**2021**	0.067	1.717	0.086	Not clustered
**2022**	–	–	–	–
**2023**	0.061	1.977	0.048	Clustered

Local spatial autocorrelation analysis further identified localized clustering patterns for overall echinococcosis, CE, and AE in particular years and geographic areas. Due to substantial missing values across 32 counties, data for 2022 were excluded, which did not meet the completeness threshold required for reliable spatial analysis. High–high clusters of overall echinococcosis prevalence were observed mainly in western Xizang during the analyzed years. In 2019, the high-high cluster was observed in Gaize County, shifting to Cuoqin County beginning in 2020. In contrast, low-low clusters were mainly distributed across south-central counties, with the highest concentration recorded in 2021 (Fig 2). The spatial clustering of CE prevalence closely mirrored the pattern of overall prevalence, though a notable shift in high-high clustering back to Gaize County occurred in 2021 (S3 Fig in S1 File). AE prevalence demonstrated a more discontinuous spatial pattern. Hotspots were detected in Baqing County in 2019 and in Dingqing and Biru counties in 2021. Regionally, low–low clusters were observed mainly in the south-central region of Xizang in the analyzed years (S4 Fig in S1 File).

**Fig 2:**
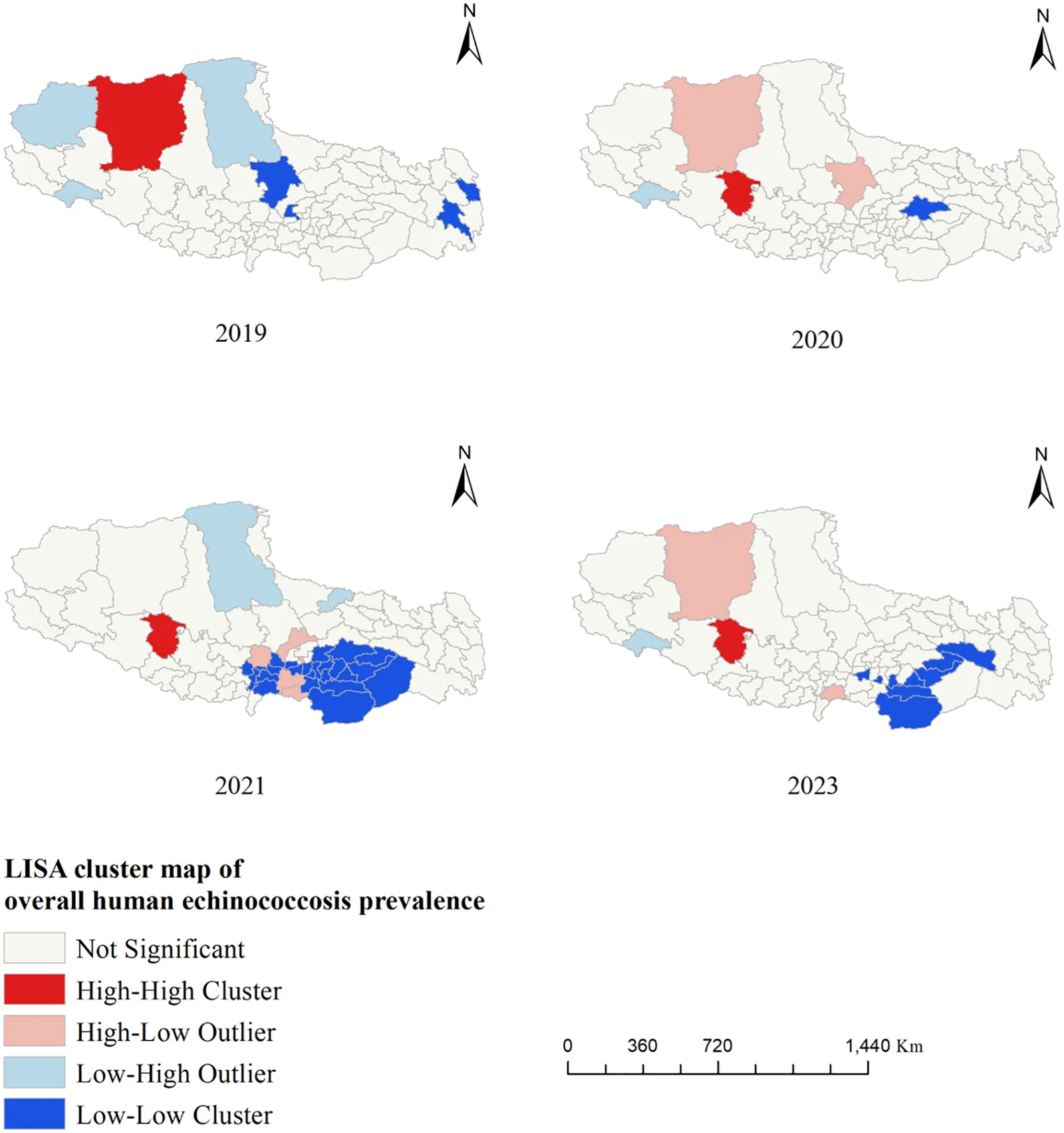
LISA cluster map of overall human echinococcosis prevalence at the county level in Xizang Autonomous Region. The map is based on the standard map with review number GS (2023) 2767 downloaded from the Standard Map Service website of the Ministry of Natural Resources (http://bzdt.ch.mnr.gov.cn/), with no modifications to the base map.

### Associations between environmental characteristics and human echinococcosis prevalence

A correlation analysis and VIF diagnostics were first conducted among candidate explanatory variables to identify and remove those exhibiting substantial multicollinearity. The diagnostics showed a strong Spearman correlation between AP and RH (|*r_s_*| = 0.72), and both AP and ME had VIF values exceeding 10 (AP = 12.81; ME = 12.77), indicating multicollinearity beyond the prespecified thresholds. Therefore, based on these diagnostics, AP and ME were excluded from further analyses (S5 and S6 Figs in S1 File). After exclusion of these two variables, multicollinearity diagnostics were repeated for the nine retained predictors (T, WS, RH, NDVI, FCP, GCP, SWP, LE, and HE). All pairwise Spearman correlation coefficients were $|r_{s}|<\text{0}.\text{7}\text{0}$, and all VIF values were <10, with the highest VIF observed for FCP (5.79). Thus, no problematic multicollinearity was identified among the retained predictors according to the prespecified criteria (S3 Table; S5 Fig in S1 File). In this study, the power parameter *p* of the Tweedie distribution was determined to be 1.35 for both the overall prevalence and CE prevalence, and 1.10 for the AE prevalence. These parameter values were subsequently used to construct the respective models (S7 Fig in S1 File).

Graphical diagnostics of the deviance residuals showed no clear systematic trends in the residual-versus-fitted plots or pronounced departures in the Q–Q plots, and no major anomalous structure was evident in the residual distributions (Figs S8–S10 in S1 File). Residual Moran’s I ranged from −0.020 to 0.030 for overall echinococcosis, from −0.030 to 0.050 for CE, and from −0.116 to −0.017 for AE. No statistically significant global spatial autocorrelation was detected in the residuals in any study year (all *P* > 0.05; S4 Table in S1 File). Basis-dimension diagnostics provided no clear evidence of insufficient basis dimensions in any of the three final models (all evaluable terms, *P* > 0.05; S5 Table in S1 File).

County-grouped five-fold cross-validation was additionally used to evaluate out-of-sample predictive performance. The RMSE and MAE were 2.16 and 0.82 for overall human echinococcosis, 1.57 and 0.66 for CE, and 0.12 and 0.02 for AE, respectively (S6 Table in S1 File).A GAMM was then fitted using overall human echinococcosis prevalence as the response variable and the remaining predictors (T, WS, RH, NDVI, FCP, GCP, SWP, LE, and HE) as smooth terms. A total of 37 candidate models were evaluated, comprising the main-effects model and 36 single-interaction models, each containing one pairwise interaction while retaining the corresponding main effects. The model with the lowest AIC (580.87) within this predefined candidate set was retained for subsequent evaluation. The final overall echinococcosis model had an adjusted R^2^ of 0.66 and explained 70.10% of the deviance (S7 Table in S1 File), indicating that a substantial proportion of the observed variation was captured in-sample. After adjustment for the other model terms, T showed a significant, approximately linear inverse association with overall human echinococcosis prevalence across the analyzed years (edf = 1.00, *F* = 7.28, *P* = 0.007; Table 3; Fig 3). None of the remaining environmental main effects reached statistical significance (all *P* > 0.05). The RH × LE interaction was statistically significant (edf = 7.10, *F* = 2.12, *P* = 0.030), indicating that the association between RH and predicted overall echinococcosis prevalence varied across levels of LE. Response-scale predictions showed a non-uniform joint pattern, with the highest predicted prevalence occurring under combinations of relatively high RH and low LE. Predicted prevalence decreased markedly toward higher LE within this high-RH region, whereas most other RH–LE combinations were associated with relatively low predicted prevalence (Fig 4).

**Table 3:** Estimated significance of smooth terms from the generalized additive mixed model of overall human echinococcosis prevalence in Xizang Autonomous Region,2019–2023.

Predictor	edf	Ref.df	*F*	*P*
**WS**	1.00	1.00	0.83	0.363
**RH**	3.30	3.70	1.76	0.091
**NDVI**	1.00	1.00	0.24	0.628
**T**	1.00	1.00	7.28	0.007
**GCP**	1.69	1.84	0.33	0.700
**SWP**	2.36	2.44	0.59	0.692
**FCP**	1.00	1.00	0.26	0.611
**LE**	1.00	1.00	1.19	0.276
**HE**	1.00	1.00	0.01	0.950
**Interaction: RH × LE**	7.10	8.38	2.12	0.030
**Spatial smooth (lon, lat)**	2.00	2.00	3.19	0.043
**County-level random effect**	42.02	66.00	1.66	< 0.001

Abbreviations: edf, estimated degrees of freedom; Ref.df, Reference degrees of freedom; WS, mean annual wind speed, RH: mean annual relative humidity; NDVI, normalized difference vegetation index; T, mean annual temperature; GCP, grassland coverage percentage; SWP, surface water coverage percentage; FCP, forest coverage percentage; LE, lowest elevation; HE, highest elevation. For the county random-effect smooth, edf reflects the model complexity associated with the penalized county-level random intercept.

**Fig 3:**
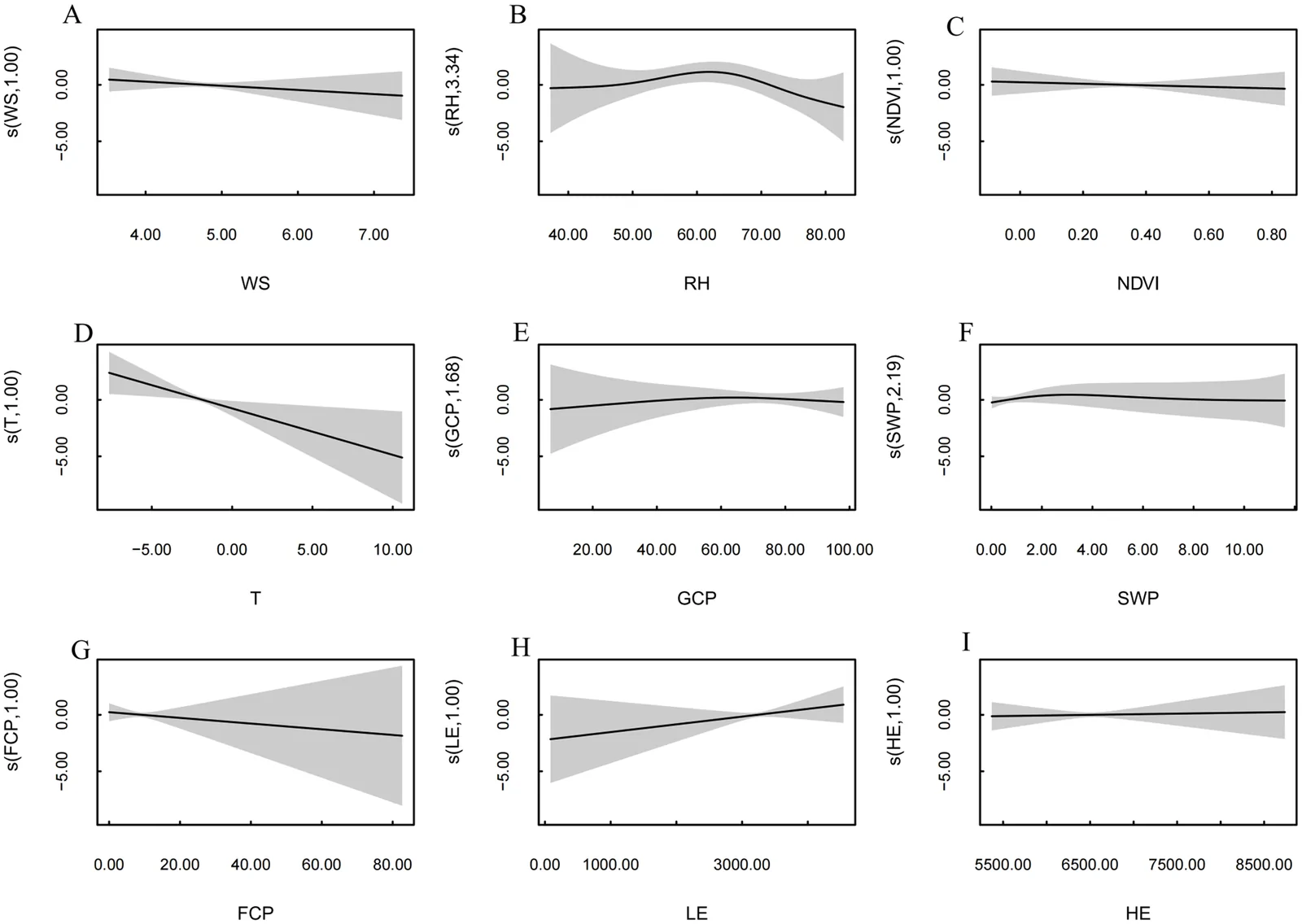
Estimated smooth functions for covariates in the GAMM of overall human echinococcosis prevalence. (A) WS: mean annual wind speed, (B) RH: mean annual relative humidity, (C) NDVI: normalized difference vegetation index, (D) T: mean annual temperature, (E) GCP: grassland coverage percentage, (F) SWP: surface water coverage percentage, (G) FCP: forest coverage percentage, (H) LE: lowest elevation, and (I) HE: highest elevation. The solid red line represents the estimated mean effect curve, and the shaded gray area indicates the 95% confidence interval.

**Fig 4:**
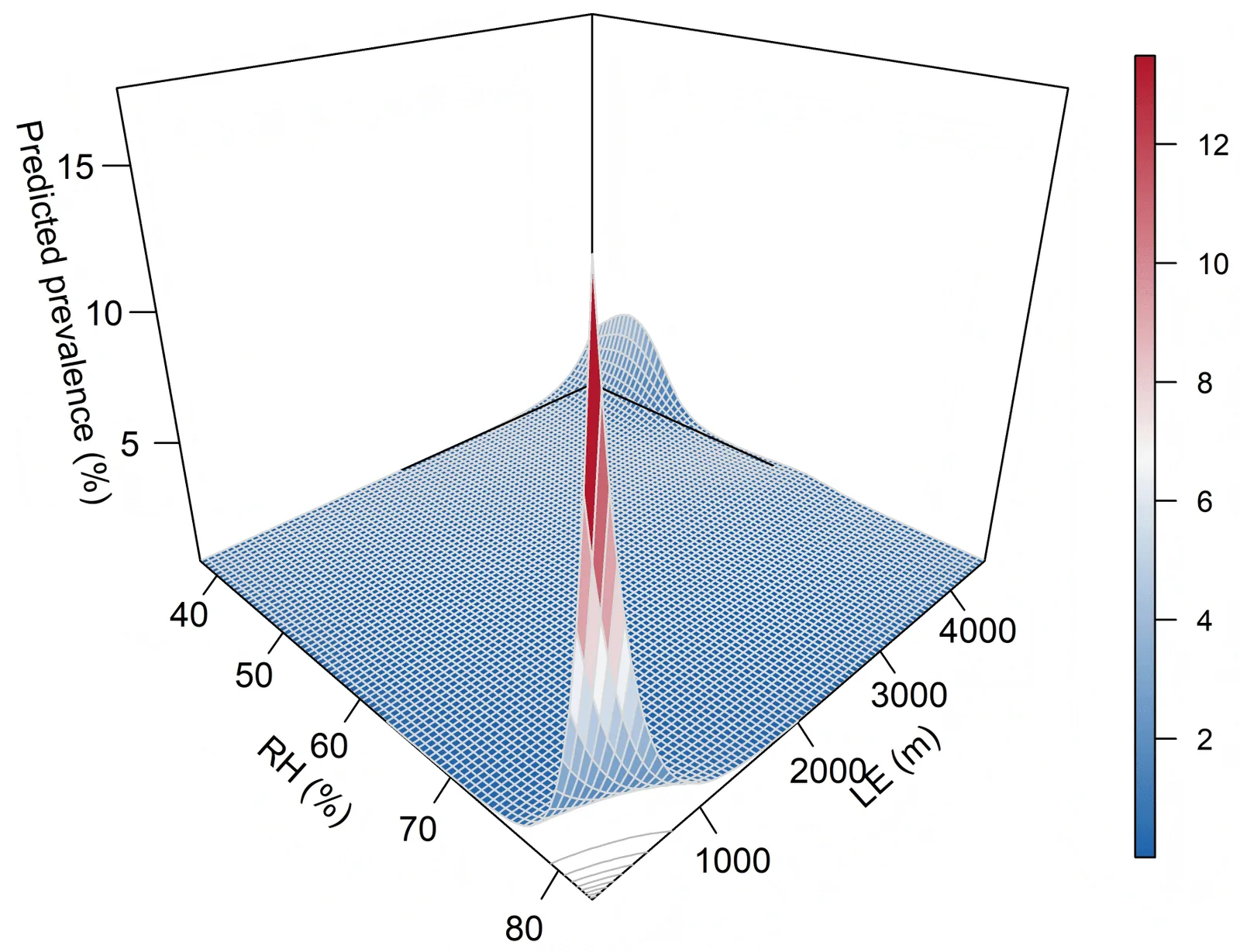
Three-dimensional predicted prevalence surface for the interaction between RH and LE in the overall human echinococcosis model. The divergent color scale represents the magnitude of predicted prevalence, with warmer colors indicating higher predicted values and cooler colors indicating lower predicted values.

Using the same retained environmental predictor set and the same model-building and candidate-model comparison procedures as for overall echinococcosis, a separate GAMM was fitted for CE. The model with the lowest AIC (576.80) within this predefined candidate set was retained for subsequent evaluation. The final CE model had an adjusted R^2^ of 0.66 and explained 70.90% of the deviance (S8 Table in S1 File), indicating that a substantial proportion of the observed variation was captured in-sample. After adjustment for the other model terms, RH was significantly associated with CE prevalence (edf = 4.67, *F* = 2.17, *P* = 0.034; S9 Table in S1 File). Its smooth curve showed a non-monotonic pattern, with local peaks at approximately 45% and 65% RH and a trough around 75% (S11 Fig in S1 File). None of the remaining environmental main effects were statistically significant (all *P* > 0.05). Importantly, the T × SWP interaction was statistically significant (edf = 3.11, *F* = 3.93, *P* = 0.005), indicating that the association between T and predicted CE prevalence varied across levels of SWP. Response-scale predictions showed a non-uniform joint surface: predicted CE prevalence was generally higher under lower-temperature conditions, particularly across intermediate levels of SWP, and declined as T increased; at higher T, predicted prevalence remained relatively low across most of the SWP range (S12 Fig in S1 File). For AE, a parsimonious GAMM was fitted using three prespecified environmental predictors (T, GCP, and LE), with no interaction terms included. The revised model had an adjusted R^2^ of 0.75 and explained 79.82% of the deviance, indicating substantial in-sample fit. Among the three prespecified environmental predictors, only LE was significantly associated with AE prevalence after adjustment for the other model terms (edf = 1.00, *F* = 4.94, *P* = 0.027; S10 Table in S1 File). The estimated smooth showed an approximately linear positive association, with higher LE associated with higher AE prevalence (S13 Fig in S1 File).

### Sensitivity analyses

Sensitivity analyses using an alternative k-nearest-neighbor (kNN) spatial weights matrix showed that the direction of global spatial autocorrelation was largely preserved, with Global Moran’s I retaining the same direction in 11 of 12 outcome-year comparisons, although statistical significance differed in four comparisons. Local Moran’s I results were more sensitive to the weighting specification, with the kNN approach generally identifying fewer significant counties and some changes in local spatial classifications (S11–S12 Tables in S1 File).

Tweedie power sensitivity analyses showed that the principal smooth associations and interaction surfaces for overall echinococcosis and CE were broadly consistent across alternative *p* specifications. For overall echinococcosis, the primary value of p = 1.35 was compared with p = 1.25 and 1.45, and the RH × LE interaction surface remained broadly similar. For CE, similar patterns were observed across p = 1.25, 1.35, and 1.45 for the principal smooth associations and the T × SWP interaction. In contrast, the AE smooth associations changed more noticeably when the primary p = 1.10 was replaced by p = 1.20 or 1.30, indicating greater sensitivity to the Tweedie distributional specification (S13 Table in S1 File).

Leave-one-year-out analyses further indicated temporal sensitivity in the AE model. The T and LE associations generally retained their direction and approximately linear form across year exclusions, but their magnitude and statistical support varied, particularly after exclusion of 2021. For LE, the shape RMSE increased to 2.280 and the *P* value changed from 0.027 in the full model to 0.781 after excluding 2021. The GCP smooth showed greater variation in shape, with the largest discrepancy observed after exclusion of 2019 (correlation = −0.878; shape RMSE = 2.001), while remaining statistically non-significant in all analyses. Overall, the AE-specific environmental associations showed greater temporal and distributional sensitivity and should therefore be interpreted cautiously (S14 Table in S1 File).

## Discussion

Although echinococcosis remains a major public health challenge in Xizang, substantial progress has been achieved in recent years. Compared with 2016, CE was reported in all 74 counties and AE in 47 counties [9], the endemic range of echinococcosis contracted markedly during 2019–2023. In parallel, the overall prevalence declined from 1.66% in 2016 (1.45% for CE and 0.20% for AE) to an average of 0.48% during the study period (0.43% for CE and 13.92 per 100,000 for AE). These reductions occurred during the implementation of an integrated control strategy in Xizang since 2017, which prioritized source control while combining intermediate host management and population-based screening and treatment [32].

Despite this overall decline, marked spatial heterogeneity was observed across Xizang during the study period. CE prevalence closely mirrored the spatial pattern of overall echinococcosis, with high-prevalence areas concentrated in the pastoral regions of northern and western Xizang and low-prevalence areas mainly distributed in the southeastern agricultural zone, which is consistent with earlier findings [10]. In contrast, AE showed a more focal spatial distribution, with higher-prevalence areas concentrated mainly in northeastern Xizang and localized high–high clusters identified there in selected years. Prevalence maps and spatial clustering analyses therefore showed broadly concordant geographic patterns within the surveillance data. Sensitivity analyses using an alternative spatial-weight specification generally preserved the direction of the principal spatial patterns, although the statistical significance of some global and local spatial associations varied. This suggests that the broad geographic contrasts were relatively consistent, whereas the identification of statistically significant clustering was partly dependent on how spatial neighbourhoods were defined. These patterns should nevertheless be interpreted with consideration of potential variation in the representativeness of sampled villages. The differing distributions of CE and AE therefore reflect subtype-specific spatial heterogeneity rather than directly indicating differences in transmission intensity.

At the county level, the GAMM analyses identified distinct ecological associations between natural environmental characteristics and overall human echinococcosis prevalence in Xizang. T showed an approximately linear inverse association with prevalence. This finding is consistent with previous large-scale studies in China reporting negative associations between temperature and human echinococcosis or CE prevalence [17,33,34]. A recent national analysis further showed that model-predicted infection risk in animal hosts increased under relatively humid and low-temperature conditions, while human CE prevalence was positively and nonlinearly associated with predicted infection risk in dogs and cattle [35]. These findings suggest that the observed temperature association may reflect broader environment–host–human transmission contexts rather than a direct effect of temperature on human infection. A significant nonlinear interaction between RH and LE was also identified, despite the absence of clear independent associations for either variable. Response-scale predictions showed that prevalence was highest under combinations of relatively high RH and low LE, with predicted prevalence decreasing as LE increased under higher-humidity conditions. Previous studies in western China have likewise identified relative humidity, elevation, precipitation, grassland characteristics, and water accessibility as environmental characteristics associated with the spatial distribution of human echinococcosis [15,18,33]. In Xizang specifically, precipitation, elevation, water accessibility, and dog and yak populations have also been associated with CE prevalence [18]. The RH × LE interaction therefore may represent a composite ecological setting rather than a simple independent effect of either humidity or elevation. At the county level, such environmental conditions may coincide with differences in host distribution, livestock activity, water availability, vegetation, settlement patterns, and opportunities for human–animal contact. This interpretation is also consistent with county-level studies showing that natural environmental, human, and host-related factors jointly contribute to the spatial variation of CE prevalence on the Qinghai–Tibet Plateau [15]. Accordingly, RH and LE should be interpreted as ecological indicators of broader transmission environments rather than direct determinants of prevalence. In Xizang, lower-elevation valleys often concentrate human settlements, livestock activity, water access, and human–animal contact, while humidity may also reflect vegetation and habitat conditions relevant to host communities. LE was retained after multicollinearity screening because it met the prespecified criteria and may therefore serve as an ecologically interpretable indicator of these lower-elevation inhabited environments [36], rather than being considered intrinsically superior to mean elevation. Because key host, behavioral, and transmission-related factors were not directly measured, the observed associations should be interpreted as ecological correlates rather than evidence of specific causal mechanisms.

For CE, the association with RH was distinctly nonlinear, indicating that ambient moisture conditions may relate to CE prevalence in a non-monotonic manner rather than through a simple linear gradient. This finding is broadly consistent with previous ecological studies reporting associations of precipitation, humidity-related conditions, and landscape characteristics with human echinococcosis [11,12,37]. The interaction between T and SWP further suggests that water-related landscape conditions may not have a uniform association with CE prevalence across different thermal environments. The higher predicted prevalence under colder conditions and the greater variation across SWP levels at lower temperatures indicate that the ecological relevance of surface water may depend on the broader climatic context. Previous studies have similarly linked human CE prevalence with temperature, precipitation, water-related landscape features, and other environmental characteristics [12,13,15,37]. In the dog–livestock transmission cycle of CE, such climatic and landscape conditions may coincide with differences in grazing patterns, livestock distribution, dog access to livestock, human settlement and water-use practices, and opportunities for human–animal contact [2,3,11,38]. SWP may therefore be better interpreted as an ecological indicator of environments where water availability, pastoral activity, host distribution, and human exposure co-occur, rather than as a direct causal factor. Likewise, the T × SWP interaction may reflect the combined ecological context in which these processes operate. Because host abundance and infection status, dog-management practices, livestock movements, and human behavioral exposure were not directly measured, these associations should be interpreted as county-level ecological correlates rather than evidence of a specific causal transmission mechanism.

AE showed a distinct environmental association pattern. In the simplified model, LE was positively and approximately linearly associated with AE prevalence. Previous ecological studies have also reported associations between elevation and human AE occurrence on the Qinghai–Tibet Plateau and in other endemic settings [14,16]. At the county level, LE may reflect broader ecological conditions associated with host habitats and transmission settings rather than a direct effect of elevation itself. The transmission ecology of *Echinococcus multilocularis* on the Tibetan Plateau involves complex interactions among definitive hosts, small-mammal intermediate hosts, landscape conditions, and human activities [38,39]. Given the limited number of AE cases and their marked concentration in 2021, AE-specific environmental associations were more sensitive to the Tweedie power specification and leave-one-year-out analyses. Although the general direction of the LE association was broadly maintained, its magnitude and statistical support varied, particularly when 2021 was omitted; these findings should therefore be regarded as exploratory and require confirmation in larger, more temporally balanced datasets.

Temporal trends further supported this distinction. While CE prevalence closely tracked the overall echinococcosis trend and peaked in 2020, AE prevalence peaked in 2021, indicating distinct temporal patterns between the two subtypes. Factors such as deforestation, changes in rodent populations, or the distribution of large dog breeds may contribute to AE dynamics [40]. Surveillance activities in 2022 occurred during a period of substantial COVID-19-related disruption in Xizang [41], and usable county-level echinococcosis surveillance data were unavailable for 32 of the 74 counties. Accordingly, temporal changes around 2022–2023 should be interpreted cautiously. Because comparable information on screening volume, the timing of screening suspension and resumption, and case-detection processes was unavailable, we could not determine whether the apparent increase in 2023 reflected a true epidemiological change or differences in surveillance completeness and case ascertainment.

Several limitations should be acknowledged. First, the analysis was conducted at the county level, and county centroid coordinates were used to represent spatial location in the GAMMs. This approach captures broad spatial variation among counties but cannot represent potentially substantial environmental and epidemiological heterogeneity within individual counties, particularly given the large geographic extent of some counties in Xizang. County-level aggregation may also introduce uncertainty related to the modifiable areal unit problem (MAUP). Accordingly, the observed environmental associations and spatial patterns should be interpreted as county-level ecological findings and should not be extrapolated directly to finer spatial scales such as townships or villages, or to individual-level relationships, because such extrapolation may lead to ecological fallacy. Second, the models focused on natural environmental characteristics and did not include several potentially important host-, human-, behavioral-, socioeconomic-, and intervention-related factors, such as dog density and infection status, livestock density and infection status, small-mammal host abundance or infection status relevant to AE, pastoral practices, human–animal contact patterns, population mobility, socioeconomic conditions, dog-management practices, and deworming coverage. The absence of these variables may have resulted in residual confounding. Consequently, some of the observed environmental associations may partly reflect or serve as proxies for unmeasured host–human–environment interactions rather than direct environmental effects. Third, although the surveillance system used a standardized random cluster-sampling protocol, specified a minimum of 1,000 individuals screened per surveillance site, and applied a uniform diagnostic standard (WS 257–2006), surveillance was conducted in selected village clusters rather than through complete enumeration of the county population. Therefore, within-county heterogeneity and differences in the composition of sampled villages may affect the representativeness of county-level surveillance prevalence. Moreover, because surveillance villages were not eligible for reselection within five years, different village clusters contributed to surveillance estimates across years, potentially introducing temporal variation unrelated to true changes in county-wide prevalence. Residual differences in participation, healthcare accessibility, and operational case ascertainment may also remain. Consequently, part of the observed spatial and temporal heterogeneity may reflect sampling and surveillance-related variation in addition to underlying epidemiological variation. Fourth, the AE-specific analysis was additionally constrained by the small number of detected cases and their marked temporal concentration, with 53 of the 71 cases detected in 2021. Although a parsimonious model was used to reduce model complexity, leave-one-year-out analyses indicated that some AE-specific associations, particularly their estimated magnitude and statistical precision, were sensitive to the exclusion of individual years, especially 2021. These findings should therefore be interpreted cautiously and regarded as exploratory. Finally, the findings reflect the unique high-altitude ecological context of Xizang and should be generalized to regions with different ecological and climatic conditions with caution.

## Conclusion

This study identified marked county-level spatial heterogeneity in human echinococcosis in Xizang, with distinct geographic patterns for CE and AE. CE accounted for most detected cases and was more widely distributed in western and northern Xizang, with high–high clusters occurring mainly in the west, whereas AE showed a more focal distribution, with localized clustering in northeastern Xizang. Climatic, topographic, and landscape characteristics showed subtype-specific ecological associations with prevalence, including nonlinear and interactive patterns. The AE-specific findings were less stable in sensitivity analyses and should therefore be considered exploratory. These findings represent county-level ecological associations rather than direct environmental effects on transmission. Environmental information may complement host, population, behavioral, surveillance, and intervention-related data in identifying areas warranting enhanced surveillance and further investigation. Future studies integrating host ecology, human behavior, socioeconomic conditions, and intervention-related factors are needed to clarify the processes underlying these spatial patterns and to inform One Health prevention and control.

## Supporting information

S1 File Supplementary tables and figures for the analysis of county-level spatial heterogeneity and environmental correlates of human echinococcosis in Xizang, China. (DOC)
