# Supplementary material for: County-level spatial heterogeneity and environmental correlates of human echinococcosis in Xizang, China
Source: PLoS One. 2026 Nov 10;21(11):e0362680. doi: 10.1371/journal.pone.0362680 (PMC13750649; doi:10.1371/journal.pone.0362680)
Supplement: Supplementary file 1 [file pone.0362680.s001.doc]

| **S1 Table. Results of global spatial autocorrelation for human CE prevalence in Xizang Autonomous Region, 2019–2023.** | | | | |
| --- | --- | --- | --- | --- |
| **Year** | **Moran's I** | **Z-Score** | ***P*** | **Pattern** |
| **2019** | 0.082 | 2.033 | 0.042 | Clustered |
| **2020** | 0.01 | 0.626 | 0.531 | Not clustered |
| **2021** | 0.087 | 2.149 | 0.032 | Clustered |
| **2022** | - | - | - | - |
| **2023** | 0.058 | 1.900 | 0.058 | Not clustered |

| **S2 Table. Results of global spatial autocorrelation for human AE prevalence in Xizang Autonomous Region, 2019–2023.** | | | | |
| --- | --- | --- | --- | --- |
| **Year** | **Moran's I** | **Z-Score** | ***P*** | **Pattern** |
| **2019** | 0.068 | 2.004 | 0.045 | Clustered |
| **2020** | 0.008 | 0.681 | 0.496 | Not clustered |
| **2021** | 0.032 | 2.077 | 0.038 | Clustered |
| **2022** | - | - | - | - |
| **2023** | 0.023 | 0.929 | 0.353 | Not clustered |

| **S3 Table. Variance inflation factors of environmental predictors before and after multicollinearity screening.** | | |
| --- | --- | --- |
| **Predictor** | **VIF before exclusion** | **VIF after exclusion** |
| **T** | 6.69 | 2.89 |
| **WS** | 2.26 | 1.72 |
| **RH** | 5.47 | 3.82 |
| **AP** | 12.81 | — |
| **NDVI** | 2.63 | 2.48 |
| **FCP** | 7.80 | 5.79 |
| **GCP** | 4.16 | 4.00 |
| **SWP** | 1.56 | 1.50 |
| **LE** | 4.52 | 4.51 |
| **ME** | 12.77 | — |
| **HE** | 3.05 | 2.44 |

| **S4 Table . Residual spatial autocorrelation diagnostics for the final generalized additive mixed models** | | | | | | |
| --- | --- | --- | --- | --- | --- | --- |
| **Outcome** | **Year** | **No. of counties (N)** | **Moran’s I** | **Expected Moran’s I** | **Z-Score** | ***P*** |
| **Overall echinococcosis** | 2019 | 74 | -0.020 | -0.010 | -0.080 | 0.938 |
|  | 2020 | 74 | 0.020 | -0.010 | 0.400 | 0.690 |
|  | 2021 | 74 | 0.030 | -0.010 | 0.580 | 0.561 |
|  | 2023 | 74 | 0.030 | -0.010 | 0.640 | 0.520 |
| **CE** | 2019 | 74 | -0.030 | -0.010 | -0.250 | 0.806 |
|  | 2020 | 74 | 0.010 | -0.010 | 0.280 | 0.777 |
|  | 2021 | 74 | -0.020 | -0.010 | -0.100 | 0.916 |
|  | 2023 | 74 | 0.050 | -0.010 | 0.820 | 0.410 |
| **AE** | 2019 | 74 | -0.116 | -0.014 | -1.501 | 0.133 |
|  | 2020 | 74 | -0.044 | -0.014 | -0.478 | 0.633 |
|  | 2021 | 74 | -0.017 | -0.014 | -0.047 | 0.963 |
|  | 2023 | 74 | -0.033 | -0.014 | -0.317 | 0.751 |

| **S5 Table . Basis-dimension diagnostic results for smooth terms in the final GAMMs for overall echinococcosis, CE, and AE.** | | | | | |
| --- | --- | --- | --- | --- | --- |
| **Model** | **Smooth term** | **k′** | **edf** | **k-index** | **P-value** |
| **Overall echinococcosis** | s(WS) | 5.00 | 1.00 | 1.05 | 0.990 |
|  | s(RH) | 5.00 | 3.30 | 0.81 | 0.130 |
|  | s(NDVI) | 5.00 | 1.00 | 0.94 | 0.550 |
|  | s(T) | 5.00 | 1.00 | 1.02 | 0.970 |
|  | s(GCP) | 5.00 | 1.69 | 1.12 | 1.000 |
|  | s(SWP) | 5.00 | 2.36 | 1.10 | 1.000 |
|  | s(FCP) | 5.00 | 1.00 | 1.13 | 1.000 |
|  | s(LE) | 5.00 | 1.00 | 1.11 | 1.000 |
|  | s(HE) | 5.00 | 1.00 | 1.20 | 1.000 |
|  | ti(RH, LE) | 25.00 | 7.10 | 0.86 | 0.110 |
|  | s(lon, lat) | 29.00 | 2.00 | 1.15 | 1.000 |
|  | s(County) | 74.00 | 42.02 | — | — |
| **CE** | s(WS) | 5.00 | 1.00 | 1.04 | 0.990 |
|  | s(RH) | 5.00 | 4.07 | 0.83 | 0.070 |
|  | s(NDVI) | 5.00 | 1.88 | 0.96 | 0.800 |
|  | s(T) | 5.00 | 2.75 | 1.01 | 0.950 |
|  | s(GCP) | 5.00 | 1.79 | 1.17 | 1.000 |
|  | s(SWP) | 5.00 | 2.45 | 1.13 | 1.000 |
|  | s(FCP) | 5.00 | 1.00 | 1.17 | 1.000 |
|  | s(LE) | 5.00 | 2.49 | 1.14 | 1.000 |
|  | s(HE) | 5.00 | 1.00 | 1.23 | 1.000 |
|  | ti(T, SWP) | 25.00 | 3.11 | 1.03 | 1.000 |
|  | s(lon, lat) | 29.00 | 2.00 | 1.14 | 1.000 |
|  | s(County) | 74.00 | 37.95 | — | — |
| **AE** | s(T) | 3.00 | 1.00 | 0.86 | 0.240 |
|  | s(GCP) | 3.00 | 1.00 | 1.05 | 1.000 |
|  | s(LE) | 3.00 | 1.00 | 1.03 | 0.980 |
|  | s(lon, lat) | 5.00 | 2.00 | 1.07 | 1.000 |
|  | s(County) | 74.00 | 17.60 | — | — |

| **S6 Table. Predictive performance of the final generalized additive mixed models based on county-grouped five-fold cross-validation.** | | |
| --- | --- | --- |
| **Outcome** | **RMSE** | **MAE** |
| Overall human echinococcosis | 2.16 | 0.82 |
| Cystic echinococcosis (CE) | 1.57 | 0.66 |
| Alveolar echinococcosis (AE) | 0.12 | 0.02 |

| **S7 Table. Goodness-of-fit of the baseline model and the top 10 optimized models for overall human prevalence.** | | | | |
| --- | --- | --- | --- | --- |
| **Model** | **Interraction** | **AIC** | **Adjusted R²** | **Deviance_explained (%)** |
| **main-effects model** |  | 614.46 | 0.53 | 65.85 |
| **model 1** | RH + LE | 579.72 | 0.66 | 70.10 |
| **model 2** | WS + RH | 594.41 | 0.61 | 60.90 |
| **model 3** | T + SWP | 595.53 | 0.56 | 56.00 |
| **model 4** | RH + SWP | 596.61 | 0.64 | 63.60 |
| **model 5** | RH + NDVI | 599.81 | 0.61 | 60.50 |
| **model 6** | WS + FCP | 600.29 | 0.58 | 58.10 |
| **model 7** | SWP + LE | 608.20 | 0.54 | 54.00 |
| **model 8** | RH + T | 609.58 | 0.60 | 60.10 |
| **model 9** | WS + GCP | 609.68 | 0.53 | 53.40 |
| **model 10** | WS + HE | 610.25 | 0.54 | 53.50 |

| **S8 Table. Goodness-of-fit of the baseline model and the top 10 optimized models for CE prevalence.** | | | | |
| --- | --- | --- | --- | --- |
| **Model** | **Interraction** | **AIC** | **Adjusted R²** | **Deviance_explained (%)** |
| **main-effects model** |  | 606.23 | 0.53 | 66.09 |
| **model 1** | T + SWP | 574.67 | 0.66 | 70.64 |
| **model 2** | RH + LE | 575.64 | 0.57 | 70.02 |
| **model 3** | RH + SWP | 577.08 | 0.67 | 71.83 |
| **model 4** | WS + RH | 583.93 | 0.61 | 70.19 |
| **model 5** | WS + FCP | 589.13 | 0.58 | 68.24 |
| **model 6** | RH + NDVI | 592.75 | 0.61 | 69.49 |
| **model 7** | GCP + HE | 600.05 | 0.57 | 68.13 |
| **model 8** | SWP + LE | 600.64 | 0.54 | 66.12 |
| **model 9** | SWP + FCP | 602.83 | 0.53 | 66.21 |
| **model 10** | RH + T | 574.67 | 0.57 | 70.02 |

| **S9 Table Estimated smooth functions for covariates in the GAMM of human CE prevalence in Xizang Autonomous Region, 2019–2023.** | | | | |
| --- | --- | --- | --- | --- |
| **Predictor** | **edf** | **Ref.df** | **F** | **P** |
| **WS** | 1.00 | 1.00 | 0.86 | 0.354 |
| **RH** | 4.67 | 4.88 | 2.17 | 0.034 |
| **NDVI** | 1.88 | 2.19 | 0.86 | 0.490 |
| **T** | 2.75 | 3.22 | 2.48 | 0.052 |
| **GCP** | 1.79 | 1.95 | 0.76 | 0.510 |
| **SWP** | 2.45 | 2.58 | 1.48 | 0.169 |
| **FCP** | 1.00 | 1.00 | 0.07 | 0.798 |
| **LE** | 2.49 | 2.66 | 0.93 | 0.278 |
| **HE** | 1.00 | 1.00 | 0.04 | 0.851 |
| **Interaction: T × SWP** | 3.11 | 3.64 | 3.93 | 0.005 |
| **Spatial smooth (lon, lat)** | 2.00 | 2.00 | 0.53 | 0.588 |
| **County-level random effect** | 37.95 | 66.00 | 1.45 | < 0.001 |

| **S10 Table. Estimated smooth functions for covariates in the GAMM of human AE prevalence in Xizang Autonomous Region, 2019–2023.** | | | | |
| --- | --- | --- | --- | --- |
| **Predictor** | **edf** | **Ref.df** | ***F*** | ***P*** |
| **T** | 1.00 | 1.00 | 3.18 | 0.076 |
| **GCP** | 1.00 | 1.00 | 0.23 | 0.635 |
| **LE** | 1.00 | 1.00 | 4.94 | 0.027 |
| **Spatial smooth (lon, lat)** | 2.00 | 2.00 | 3.59 | 0.029 |
| **County-level random effect** | 17.64 | 69.00 | 0.83 | < 0.001 |

| **S11 Table . Sensitivity of Global Moran’s I to alternative spatial-weights specifications.** | | | | | | | |
| --- | --- | --- | --- | --- | --- | --- | --- |
| **Outcome** | **Year** | **Moran_I_ Queen** | ***Z*_Queen** | ***P*_Queen** | **Moran_I_ kNN** | ***Z*_kNN** | ***P*_kNN** |
| **Overall** | 2019 | 0.049 | 1.303 | 0.193 | 0.012 | 0.408 | 0.684 |
| **Overall** | 2020 | 0.009 | 0.582 | 0.561 | 0.002 | 0.32 | 0.749 |
| **Overall** | 2021 | 0.067 | 1.717 | 0.086 | 0.118 | 2.158 | 0.031 |
| **Overall** | 2023 | 0.061 | 1.977 | 0.048 | 0.077 | 1.83 | 0.067 |
| **CE** | 2019 | 0.082 | 2.033 | 0.042 | 0.024 | 0.614 | 0.539 |
| **CE** | 2020 | 0.01 | 0.626 | 0.531 | 0.004 | 0.363 | 0.716 |
| **CE** | 2021 | 0.087 | 2.149 | 0.032 | 0.09 | 1.706 | 0.088 |
| **CE** | 2023 | 0.058 | 1.9 | 0.058 | 0.072 | 1.731 | 0.083 |
| **AE** | 2019 | 0.068 | 2.004 | 0.045 | 0.094 | 2.038 | 0.042 |
| **AE** | 2020 | 0.008 | 0.681 | 0.496 | 0.051 | 1.585 | 0.113 |
| **AE** | 2021 | 0.032 | 2.077 | 0.038 | 0.103 | 4.083 | <0.001 |
| **AE** | 2023 | 0.023 | 0.929 | 0.353 | -0.025 | -0.221 | 0.825 |

| **S12 Table . Concordance of significant Local Moran’s I patterns under alternative spatial-weights specifications.** | | | | | |
| --- | --- | --- | --- | --- | --- |
| **Outcome** | **Year** | **Queen n** | **kNN n** | **Concordant patterns** | **Discordant classifications** |
| **Overall** | 2019 | 8 | 5 | Gaize (HH); Ritu (LH) | None |
| **Overall** | 2020 | 5 | 2 | Pulan (LH) | None |
| **Overall** | 2021 | 28 | 4 | Milin (LL); Namulin (HL); Shuanghu (LH) | None |
| **Overall** | 2023 | 13 | 5 | Pulan (LH) | Cuona: LL → HH |
| **CE** | 2019 | 8 | 3 | Gaize (HH); Ritu (LH) | None |
| **CE** | 2020 | 4 | 3 | Pulan (LH) | None |
| **CE** | 2021 | 25 | 3 | Milin (LL); Shuanghu (LH) | None |
| **CE** | 2023 | 12 | 5 | Pulan (LH) | Cuona: LL → HH |
| **AE** | 2019 | 11 | 3 | Bianba (LH); Suo (LH) | None |
| **AE** | 2020 | 6 | 1 | Suo (LH) | None |
| **AE** | 2021 | 24 | 5 | Suo (LH) | Anduo: HL → HH; Nimu: LL → LH |
| **AE** | 2023 | 7 | 1 | None | None |

| **S13 Table . Sensitivity of the GAMM results to alternative Tweedie power parameters.** | | | | | |
| --- | --- | --- | --- | --- | --- |
| **Outcome** | **Primary p** | **Alternative p values** | **Principal smooth associations** | **Interaction pattern** | **Overall interpretation** |
| **Overall echinococcosis** | 1.35 | 1.25, 1.45 | Broadly similar | RH × LE: broadly similar | Main conclusions stable |
| **CE** | 1.35 | 1.25, 1.45 | Broadly similar | T × SWP: broadly similar | Main conclusions partly sensitive |
| **AE** | 1.10 | 1.20, 1.30 | Broadly changed | — | Main conclusions partly sensitive |

| **S14 Table. Leave-one-year-out sensitivity analysis of environmental smooth associations in the AE model.** | | | | | | |
| --- | --- | --- | --- | --- | --- | --- |
| **Variable** | **Analysis** | **edf** | **F** | **P value** | **Correlation with full-model smooth** | **Shape RMSE** |
| **T** | Full model | 1.00 | 3.18 | 0.075 | Reference | Reference |
|  | Exclude 2019 | 1.00 | 0.81 | 0.370 | 1.00 | 0.05 |
|  | Exclude 2020 | 1.00 | 4.64 | 0.032 | 1.00 | 0.06 |
|  | Exclude 2021 | 1.00 | 0.81 | 0.371 | 1.00 | 1.48 |
|  | Exclude 2023 | 1.00 | 1.78 | 0.184 | 1.00 | 0.20 |
| **GCP** | Full model | 1.00 | 0.23 | 0.635 | Reference | Reference |
|  | Exclude 2019 | 1.75 | 0.22 | 0.799 | -0.88 | 2.00 |
|  | Exclude 2020 | 1.00 | 0.11 | 0.741 | 1.00 | 0.15 |
|  | Exclude 2021 | 1.00 | 0.10 | 0.753 | 1.00 | 0.30 |
|  | Exclude 2023 | 1.49 | 0.27 | 0.637 | 0.43 | 0.35 |
| **LE** | Full model | 1.00 | 4.94 | 0.027 | Reference | Reference |
|  | Exclude 2019 | 1.00 | 1.99 | 0.160 | 1.00 | 2.51 |
|  | Exclude 2020 | 1.00 | 5.76 | 0.017 | 1.00 | 0.12 |
|  | Exclude 2021 | 1.00 | 0.08 | 0.781 | 1.00 | 2.28 |
|  | Exclude 2023 | 1.00 | 2.82 | 0.095 | 1.00 | 0.50 |

**S1 Fig. Spatial distribution of human CE prevalence at the county level in Xizang Autonomous Region.**

The map is based on the standard map with review number GS (2023) 2767 downloaded from the Standard Map Service website of the Ministry of Natural Resources (<http://bzdt.ch.mnr.gov.cn/>), with no modifications to the base map. Areas shown in grey and labeled “Not known” indicate counties without usable surveillance data. In 2022, this applied to 32 of 74 counties (43.2%).

**S2 Fig. Spatial distribution of human AE prevalence at the county level in Xizang Autonomous Region.**

The map is based on the standard map with review number GS (2023) 2767 downloaded from the Standard Map Service website of the Ministry of Natural Resources (<http://bzdt.ch.mnr.gov.cn/>), with no modifications to the base map. Areas shown in grey and labeled “Not known” indicate counties without usable surveillance data. In 2022, this applied to 32 of 74 counties (43.2%).

**S3 Fig. LISA cluster map of human CE prevalence at the county level in Xizang Autonomous Region.**

The map is based on the standard map with review number GS (2023) 2767 downloaded from the Standard Map Service website of the Ministry of Natural Resources (<http://bzdt.ch.mnr.gov.cn/>), with no modifications to the base map.

**S4 Fig. LISA cluster map of human AE prevalence at the county level in Xizang Autonomous Region.**

The map is based on the standard map with review number GS (2023) 2767 downloaded from the Standard Map Service website of the Ministry of Natural Resources (<http://bzdt.ch.mnr.gov.cn/>), with no modifications to the base map.

**S5 Fig. Spearman correlation matrices of environmental predictors before and after multicollinearity screening.**
(A) Spearman correlations among all candidate environmental predictors before variable exclusion. (B) Spearman correlations among the nine environmental predictors retained after exclusion of AP and ME. Values within the cells represent Spearman’s rank correlation coefficients (rs).

**S6 Fig. VIF of variables.**

**S7 Fig. Log-likelihood profiles demonstrating the corresponding log-likelihoods given different values of power parameter p in the Tweedie distribution models for overall human prevalence (a), CE prevalence (b) and AE prevalence (c).**

**S8 Fig. Model diagnostics of the GAMM for overall human echinococcosis prevalence.**

**S9 Fig. Model diagnostics of the GAMM for human CE echinococcosis prevalence.**

**S10 Fig. Model diagnostics of the GAMM for human AE echinococcosis prevalence.**

**S11 Fig. Partial effects of covariates on human CE prevalence.**

(A) WS: mean annual wind speed, (B) RH: mean annual relative humidity, (C) NDVI: normalized difference vegetation index, (D) T: mean annual temperature, (E) GCP: grassland coverage percentage, (F) SWP: surface water coverage percentage, (G) FCP: forest coverage percentage, (H) LE: lowest elevation, and (I) HE: highest elevation. The solid red line represents the estimated mean effect curve, and the shaded gray area indicates the 95% confidence interval.

**S12 Fig. Three-dimensional predicted prevalence surface for the interaction between RH and SWP in the CE model.**

The divergent color scale represents the magnitude of predicted prevalence, with warmer colors indicating higher predicted values and cooler colors indicating lower predicted values.

**S13 Fig. Partial effects of covariates on human AE prevalence.**

(A) T: mean annual temperature, (B) GCP: grassland coverage percentage, (C) LE: lowest elevation. The solid red line represents the estimated mean effect curve, and the shaded gray area indicates the 95% confidence interval.
